# The Correlation Between Neonatal Intensive Care Unit Safety Culture and Quality of Care

**DOI:** 10.1097/PTS.0000000000000546

**Published:** 2018-11-08

**Authors:** Jochen Profit, Paul J. Sharek, Xin Cui, Courtney C. Nisbet, Eric J. Thomas, Daniel S. Tawfik, Henry C. Lee, David Draper, J. Bryan Sexton

**Affiliations:** From the ∗ Perinatal Epidemiology and Health Outcomes Research Unit, Division of Neonatology, Department of Pediatrics, Stanford University School of Medicine and Lucile Packard Children’s Hospital; † California Perinatal Quality Care Collaborative; ‡ Center for Quality and Clinical Effectiveness, Lucile Packard Children’s Hospital; § Division of General Pediatrics, Department of Pediatrics, Stanford University, Palo Alto, California; ∥ University of Texas at Houston - Memorial Hermann Center for Healthcare Quality and Safety, University of Texas Medical School, Houston, Texas; ¶ Division of Pediatric Critical Care Medicine, Department of Pediatrics, Stanford University School of Medicine; Stanford; ∗∗ Department of Applied Mathematics and Statistics, Baskin School of Engineering University of California, Santa Cruz, California; †† Department of Psychiatry, Duke University School of Medicine; ‡‡ Duke Patient Safety Center, Duke University Health System, Durham, North Carolina.

**Keywords:** Baby-MONITOR, composite measure, safety culture, teamwork, neonatal intensive care unit, CPQCC = California Perinatal Quality Care Collaborative, HAI = healthcare-associated infection, NICU = neonatal intensive care unit, NNP = neonatal nurse practitioner, RN = registered nurse, RCP = respiratory care practitioner, SAQ = Safety Attitudes Questionnaire, VON = Vermont Oxford Network, VLBW = very low-birth-weight

## Abstract

Key validated clinical metrics are being used individually and in aggregate (Baby-MONITOR) to monitor the performance of neonatal intensive care units (NICUs). The degree to which perceptions of key components of safety culture, safety climate, and teamwork are related to aspects of NICU quality of care is poorly understood. The objective of this study was to test whether NICU performance on key clinical metrics correlates with caregiver perceptions of safety culture.

**Study Design:**

Cross-sectional study of 6253 very low-birth-weight infants in 44 NICUs. We measured clinical quality via the Baby-MONITOR and its nine risk-adjusted and standardized subcomponents (antenatal corticosteroids, hypothermia, pneumothorax, healthcare-associated infection, chronic lung disease, retinopathy screen, discharge on any human milk, growth velocity, and mortality). A voluntary sample of 2073 of 3294 eligible professional caregivers provided ratings of safety and teamwork climate using the Safety Attitudes Questionnaire. We examined NICU-level variation across clinical and safety culture ratings and conducted correlation analysis of these dimensions.

**Results:**

We found significant variation in clinical and safety culture metrics across NICUs. Neonatal intensive care unit teamwork and safety climate ratings were correlated with absence of healthcare-associated infection (*r* = 0.39 [*P* = 0.01] and *r* = 0.29 [*P* = 0.05], respectively). None of the other clinical metrics, individual or composite, were significantly correlated with teamwork or safety climate.

**Conclusions:**

Neonatal intensive care unit teamwork and safety climate were correlated with healthcare-associated infections but not with other quality metrics. Linkages to clinical measures of quality require additional research.

## Abstract 2

Supplemental digital content is available in the text.

Establishing a culture of safety in health care has been a national health policy priority. The Joint Commission includes the establishment of a culture of safety as a critical component for achieving highly reliable and safe care^1^ and requires hospitals to measure and monitor safety culture in an ongoing fashion.^2^ A culture of safety has been defined as “individual and group values, attitudes, perceptions, competencies, and patterns of behavior that determine the commitment to, and the style and proficiency of, an organization’s health and safety management.”^3^ Maintaining a culture of safety is a strategy for preventing patient harm. Safety culture surveys assess caregiver perceptions of unit norms. In other areas of healthcare, caregiver perceptions of safety culture, especially its best-studied subdomains, safety climate and teamwork, have been shown to vary widely and were linked to clinical outcomes, including healthcare-associated infections (HAIs) and mortality.^4–8^

The Safety Attitudes Questionnaire (SAQ) was found to be psychometrically robust^9^ in the neonatal intensive care unit (NICU) and demonstrated significant variation in safety culture.^10^ Preterm infants in the NICU setting are fragile and require complex and prolonged intensive care. This makes them vulnerable to lapses in teamwork and patient safety. However, the relationship between NICU safety culture ratings and clinical metrics of quality has not been established. In addition, the evidence from other areas of healthcare demonstrates relatively weak links between safety culture and clinical outcomes,^11^ along with concerns about reporting bias.^12,13^

Neonatal intensive care is a complex and multidimensional activity, which the measurement of its quality should reflect. In previous work, we developed the Baby-MONITOR (Measure Of Neonatal InTensive care Outcomes Research), a composite indicator of quality of care provided to very low-birth-weight (VLBW, <1500 g) infants.^14^ A panel of experts selected 9 of 28 potential metrics in a modified Delphi experiment.^15^ This selection process was subsequently sanctioned by a sample of clinical neonatologists.^16^ Both groups identified the same nine clinical metrics. These metrics routinely collected by NICUs that are members of the California Perinatal Quality Care Collaborative (CPQCC) and the Vermont Oxford Network (VON), which collects data on nearly 85% of the VLBW infants born in the United States. Each measure is risk-adjusted, standardized, equally weighted, and averaged. The Baby-MONITOR has face validity^15,16^ and has been shown to be robust to variations in methods of construction.^14^ Several of the measures of the Baby-MONITOR more narrowly represent safety domains, including infections, antenatal steroids, hypothermia on admission, pneumothorax, and retinopathy screening. Others could be defined in more quality domains requiring teamwork for high performance (chronic lung disease, growth velocity, and any human milk feedings at discharge). Overlap exists between safety and quality and several measures require both behaviors for high performance.

Based on the associations of quality of care delivery with health worker assessments of safety and teamwork climate in other areas of medicine, we hypothesized that caregiver assessments of teamwork and safety climate would correlate with clinical metrics of quality for VLBW infants in the NICU setting, using the Baby-MONITOR and its individual subcomponents.

## METHODS

### Design

This cross-sectional study combined registry data routinely submitted by NICU members of the CPQCC^17^ with a voluntary sample of healthcare workers, participating in two simultaneous quality improvement initiatives, organized by the CPQCC focused on Delivery Room Management. More than 90% of NICUs in California are members of the CPQCC. We used the CPQCC clinical data to compute risk-adjusted scores for each subcomponent of the Baby-MONITOR. We then correlated these scores against health care worker assessments of teamwork and safety climate. Clinical data were obtained between January 1, 2010, and December 31, 2012, and safety culture survey data were collected between June and September 2011 from 44 participant NICUs. Thus, we selected the clinical data to cover approximately 1.5 years before and after the timing of the survey, providing a large clinical sample for estimation of quality of care delivery to VLBW infants in California NICUs.

The CPQCC assures high data quality through several mechanisms. It trains local NICU personnel to abstract clinical data. Annual training sessions help promote accuracy and uniformity in data abstraction. In addition, each record has range and logic checks both at the time of data collection and at data closeout, with auditing of records with excessive missing data. Definitions align with those specified for members of the VON.

### Sample

#### Infants

Our goal for this study was to create a relatively homogenous and unbiased sample of VLBW infants for comparison across NICUs.^15^ To ensure that patient outcomes reflected the quality of care of the NICU under observation, we excluded infants who died before 12 hours after delivery and those with severe congenital anomalies. We also restricted the analysis to infants born after 24 completed weeks of gestation to avoid systematic bias based on decisions to withhold resuscitation at the threshold of viability.^18^ We used multiyear analyses because of the small number of VLBW infants cared for in some institutions.

#### Health Care Workers

A cross-sectional anonymous survey of all NICU healthcare worker perceptions of teamwork was performed among a voluntary sample of NICUs participating in a quality improvement collaborative organized by the CPQCC.^17^ We offered to analyze and provide feedback on a survey of safety culture to all 61 NICUs who participated in the improvement initiative, 44 of which accepted. For all units, we used a paper-based version of the SAQ instrument to investigate safety and teamwork climate. Staff with a 0.5 full time equivalent or greater time commitment to the NICU for at least the four weeks before survey administration were invited to participate. Details of the survey and its administration can be found in the eAppendix, http://links.lww.com/JPS/A193.^19^ Our response rate was 62.9% (2073/3294), with a range across the 44 hospitals of 21.7% to 100% (mean [SD] = 69.7% [19.8%]).

The study was approved by the institutional review board at Stanford University.

### Metrics

#### Quality of Care

Details of the Baby-MONITOR have been published elsewhere.^14,15^ In brief, an expert panel selected nine metrics of quality for inclusion in the composite, including the following^1^: antenatal corticosteroid use,^2^ hypothermia (<36°C) during the first hour after delivery,^3^ nonsurgically induced pneumothorax,^4^ HAI,^5^ chronic lung disease (oxygen requirement at 36 weeks’ gestational age),^6^ timely eye exam (retinopathy of prematurity screening as recommended by the American Academy of Pediatrics),^7^ discharge on any human breast milk,^8^ growth velocity,^9^ and mortality during the birth hospitalization.^9^

Each of the metrics is scored so that a higher score indicates a better outcome. All metrics, except for timely eye exam as a process measure, are individually risk adjusted for severity of illness at the time of birth. To further classify NICU performance on each quality measure, we used a method developed by Draper and Gittoes.^20^ For each NICU and for each subcomponent of the Baby-MONITOR, a *z* score was computed as the observed rate minus the expected rate divided by its estimated standard error. These standardized *z* scores are approximately normally distributed with the mean of 0 and standard deviation of 1 when no quality differences are present.

#### Safety Culture

Of the several safety culture survey instruments in the literature, the SAQ is widely used and has good psychometric properties.^21^ The SAQ contains 30 items that load on the following six domains: teamwork climate, safety climate, job satisfaction, perceptions of management, stress recognition, and working conditions. Each item is rated on a five-point Likert scale ranging from 1 (disagree strongly) to 5 (agree strongly). Positions included attending physicians, fellow physicians, neonatal nurse practitioners, registered nurses (RNs), respiratory care practitioners, and others.

Safety culture scale scores were calculated at the NICU level as follows: first, we created for each scale item a binary variable that was 1 if respondents “strongly” or “slightly” agreed with the item and 0 otherwise, and then, we computed the means of these dichotomous variables.^9,21^ We call this “percentage agree” or “percentage reporting good ‘safety climate’ or ‘teamwork climate.’”

### Analyses

We used summary statistics such as frequencies, percentages, means (standard deviations), and graphs to describe demographics and our three variables: Baby-MONITOR (with subcomponents), safety climate, and teamwork climate. For each NICU, we computed *z* scores and percentiles for the Baby-MONITOR and its subcomponents, as well as percent positive rates at the scale and item level for the safety climate and teamwork scales. We then used Pearson correlation coefficient to test for correlations between the clinical and safety culture metrics.

Statistical analyses were performed using SAS (Version 9.4; SAS Institute, Inc, Cary, NC).

## RESULTS

Table 1 shows the characteristics of survey respondents and the clinical sample. A total of 6253 VLBW infants in 44 NICUs met the inclusion criteria. Of these NICUs, 10 (22.7%) were designated as regional NICUs, 28 (63.6%) as community NICUs, and 5 (11.4%) as intermediate NICUs as defined by the California Department of Healthcare Services. These designations are roughly equivalent with designations by the American Academy of Pediatrics as level 4, 3, and 2, respectively.^22^ Most respondents were RNs and female. The distribution of job positions among respondents mirrored the distribution of eligible participants across participating NICUs. Providers were quite experienced, with the largest number of respondents (643 [33%]) having worked more than 20 years in their specialty. Clinical characteristics are slightly better than all California estimates.^14^

**TABLE 1:** Description of Sample

Characteristics	Level	n	%
*Survey respondents*			
*NICU level (n = 44)*			
CCS level	Regional	10	23
	Community	28	64
	Intermediate	5	11
	Non-CCS	1	2
*Respondent level (n = 2073)*			
Female		1697	85
Typical shift	Days	894	48
	Evenings	79	4
	Nights	602	32
	Variable	293	16
Job position	Physician	204	10
	Fellow physician	31	2
	NNP	35	2
	RN	1464	72
	RCP	286	14
	Others	21	1
Working experience in specialty	<6 mo	20	1
	6–11 mo	27	1
	1–2 y	74	4
	3–4 y	192	10
	5–10 y	476	24
	11–20 y	538	27
	≥21 y	643	33
*Clinical sample (n = 6253)*			
Gestational age, wk	25–27	2134	34
	28–29	1682	27
	≥30	2437	39
Female		3091	49
Prenatal care		6057	97
Multiple gestation		1713	27
Cesarean delivery		4695	75
SGA		1144	18
Maternal age, y	≤19	578	9
	20–29	2640	42
	30–39	2588	41
	≥40	447	7
Apgar 5 min	≤3	299	5
	4–6	1047	17
	≥7	4878	78
Outborn		761	12
*Baby-MONITOR metrics of quality of care*			
No HAI		5463	91
No chronic lung disease		4302	79
Any human breast milk at discharge		3728	66
High growth velocity		2654	55
Survival		5789	94
Antenatal steroid use		4524	90
No hypothermia on admission		5598	90
No pneumothorax		6033	97
Timely retinopathy exam		3883	96

CCS, California Children's Services; Non-CCS: NICUs not certified by the California Children's Services Program; NNP, neonatal nurse practitioner; RCP, respiratory care practitioner; SGA, small for gestational age.

**FIGURE 1:**
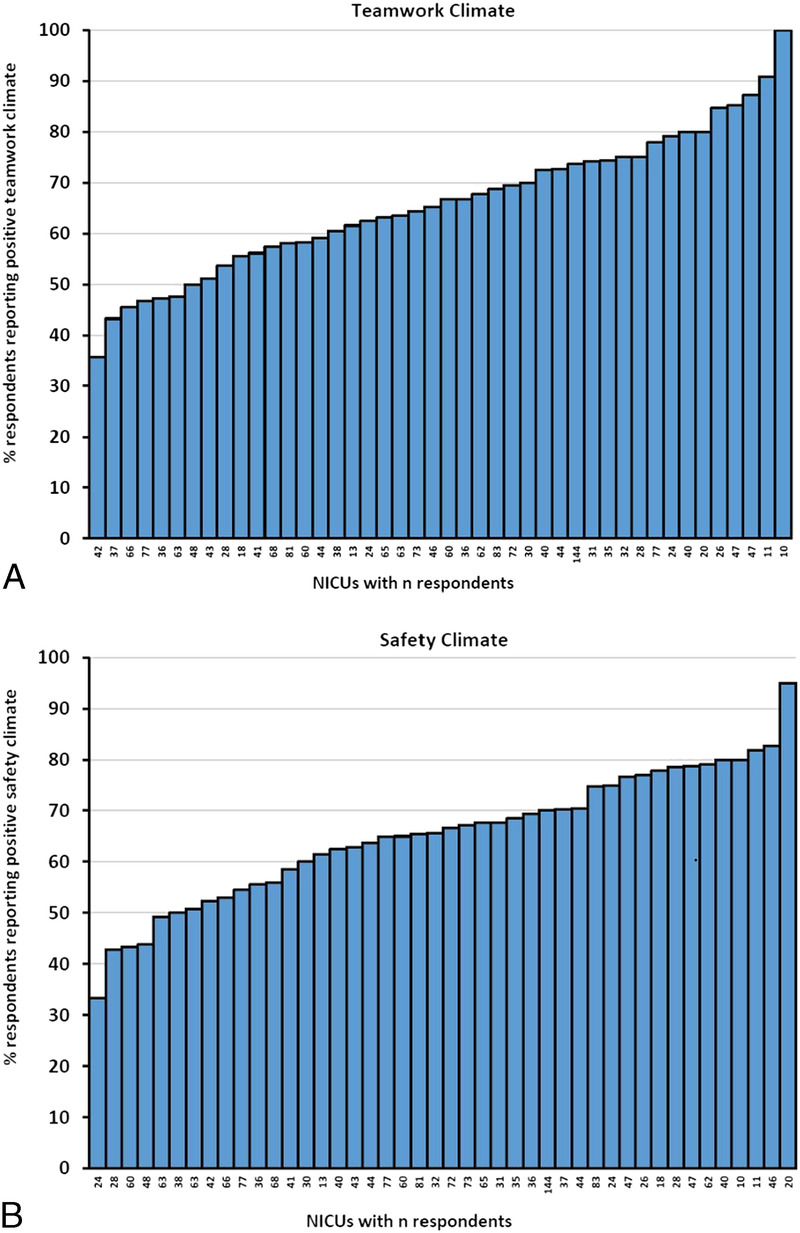
A, Percent of positive responses for the teamwork climate in 44 NICUs. Percent of positive teamwork climate is the percent of respondents responding in the “agree slightly” to “agree strongly” range across the positively worded items. B, Percent of positive responses for the safety climate in 44 NICUs. Percent of positive safety climate is the percent of respondents responding in the “agree slightly” to “agree strongly” range across the positively worded items. Numbers in x-axis, number of respondents in each NICU.

Figures 1A and B show the percent of positive responses for the teamwork and safety climate scales, respectively. The mean (SD, range) percent positive response was 77.6% (6.2, 64.9%–89.6%) for teamwork and 77.0% (5.5, 66.2%–86.6%) for safety climate. Variation between NICUs was significant for both scales (analysis of variance; *P* < 0.001). Table 2 shows the distribution of responses across all respondents for the teamwork and safety climate scales and items. For all items, the response scores of the top 4 NICUs were significantly different from the bottom 4 NICUs.

**TABLE 2:** Teamwork and Safety Climate Item Response Distributions*

	Overall	Bottom 4 NICUs	Top 4 NICUs	Difference Between the Top and Bottom 4 NICUs
	Mean (SD)	Mean (SD)	Mean (SD)	
Teamwork climate				
It is easy for personnel here to ask questions when there is something they do not understand.	83.79 (5.35)	73.43 (1.15)	91.67 (0.72)	^‡^
I have the support I need from others in this NICU to care for patients.	85.73 (4.88)	77.91 (1.73)	95.00 (2.22)	^‡^
Nurse input is well received in this NICU.	77.24 (8.35)	61.93 (3.89)	91.11 (4.27)	^‡^
In this NICU, it is difficult to speak up if I perceive a problem with patient care.	28.07 (7.45)	14.28 (3.64)	40.48 (0.63)	^‡^
Disagreements in this NICU are appropriately received.	69.07 (8.51)	55.15 (1.14)	85.06 (2.22)	^‡^
The physicians and nurses here work together as a well-coordinated team.	77.51 (10.48)	55.85 (2.16)	92.84 (1.79)	^‡^
Safety climate				
The culture in this NICU makes it easy to learn from the errors of others.	67.61 (7.80)	55.14 (1.84)	80.65 (1.93)	^‡^
Medical errors are handled appropriately in this NICU.	83.71 (6.97)	71.16 (2.66)	95.29 (3.30)	^†^
I know the proper channels to direct questions regarding patient safety in this NICU.	87.73 (4.15)	80.93 (1.11)	94.54 (1.01)	^‡^
I am encouraged by others in this NICU, to report any patient safety concerns I may have.	79.96 (5.68)	70.03 (0.51)	89.15 (1.40)	^‡^
I receive appropriate feedback about my performance.	74.32 (7.12)	62.51 (2.23)	86.30 (2.61)	^‡^
I would feel safe being treated here as a patient.	80.94 (8.20)	67.50 (0.58)	96.32 (1.68)	^§^
In this NICU, it is difficult to discuss errors.	35.04 (8.75)	16.32 (7.11)	49.12 (3.78)	^†^

*Scale score for a respondent = ((mean of the item from NICUs − 1) × 25).

^†^*P* < 0.01.

^‡^*P* < 0.001.

^§^*P* < 0.0001.

The NICU Baby-MONITOR scores ranged from −2.5 to 1.7 standard units, indicating significant and clinically meaningful variation (eFigure 1 in the eAppendix shows NICU-level variation, http://links.lww.com/JPS/A193).

eTable 1 in the eAppendix, http://links.lww.com/JPS/A193, exhibits results at the NICU level, including the percent positive responses for the teamwork and safety climate scales and the observed minus expected scores in standard deviation units and percentiles for the Baby-MONITOR and each of its subcomponents. We found significant variation in performance across the composite and its subcomponents. The widest variation of Baby-MONITOR scores and its subcomponents between the top and bottom performing NICU was found in growth velocity, with observed minus expected performance ranging from −7.3 to 10.7 standard units. By definition, a difference of 1.96 standard units implies statistical significance; these variations are large in clinical terms.

**FIGURE 2:**
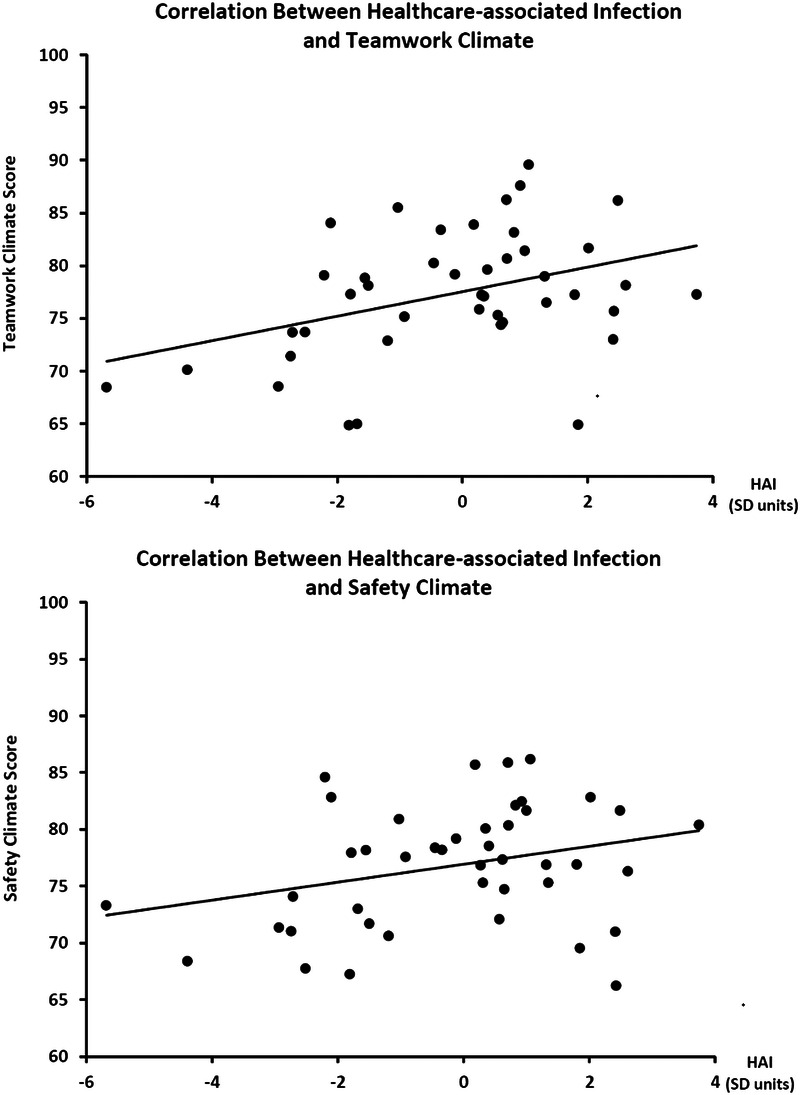
A, Correlation between HAI and teamwork climate. Each dot represents one NICU. B, Correlation between HAI and safety climate. Each dot represents one NICU. x-axis, HAI in SD units; y-axis, safety climate score.

Table 3 shows the Pearson correlation coefficients between teamwork and safety climate with the Baby-MONITOR and its subcomponents. Only HAI exhibited a statistically significant relationship (Figs. 2A, B). None of the other components of the Baby-MONITOR score or the composite were significantly correlated with teamwork and safety climate. eTables 2A and B in the eAppendix, http://links.lww.com/JPS/A193, show the correlations between teamwork climate items, safety climate items, and Baby-MONITOR and its subcomponents. Both positive and negative correlations were observed with no predominant pattern.

**TABLE 3:** Correlations Between Teamwork and Safety Climate and Baby-MONITOR and Its Subcomponents

	Teamwork Climate		Safety Climate	
	*r* (95% CI)	*P*	*r* (95% CI)	*P*
No HAI	**0.39 (0.10** to **0.61)**	**0.01**	0.29 (−0.01 to 0.55)	0.05
No chronic lung disease	0.17 (−0.13 to 0.45)	0.27	0.18 (−0.13 to 0.45)	0.25
Any human breast milk at discharge	0.02 (−0.28 to 0.32)	0.89	0.09 (−0.22 to 0.38)	0.57
High growth velocity	−0.05 (−0.35 to 0.25)	0.74	−0.20 (−0.47 to 0.10)	0.19
Survival	0.15 (−0.15 to 0.43)	0.33	0.24 (−0.06 to 0.50)	0.12
Antenatal steroid use	−0.05 (−0.36 to 0.27)	0.78	0.01 (−0.30 to 0.33)	0.93
No hypothermia on admission	−0.06 (−0.35 to 0.25)	0.72	−0.16 (−0.44 to 0.14)	0.30
No pneumothorax	0.25 (−0.05 to 0.51)	0.11	0.07 (−0.23 to 0.36)	0.63
Timely retinopathy exam	−0.07 (−0.37 to 0.23)	0.64	−0.16 (−0.44 to 0.15)	0.32
Baby-MONITOR score	0.14 (−0.17 to 0.42)	0.38	0.02 (−0.28 to 0.31)	0.92

n = 44 NICUs.

95% CI, 95% confidence interval; *r*, Pearson correlation coefficient.

## DISCUSSION

This study extends findings from the healthcare literature demonstrating substantial variability in safety culture and clinical metrics of quality and outcomes. Our findings also reinforce results from studies in adult intensive care settings,^23^ and our own previous work, which revealed links between safety or teamwork climate and infection-related outcomes. Healthcare workers in NICUs who report that “I would feel safe being treated here” work in units with lower infection rates. The reverse of this association is also true, whereby NICUs with high infection rates have fewer healthcare workers who report “It is easy to ask questions when there is something they don’t understand” or “Physicians and nurses work together as a well-coordinated team.” However, we were surprised that none of the other clinical outcomes were significantly correlated with safety or teamwork climate. Overall, our findings reflect a weaker than expected correlation of metrics of quality with teamwork and safety climate.

A priori, we did expect some metrics to yield lower correlations, particularly because some of them may be outside the direct purview of many frontline providers in the NICU (antenatal corticosteroid therapy, pneumothorax, hypothermia on admission, and timely eye exam). For example, obstetricians provide antenatal steroids, specialized delivery room teams may be accountable for pneumothoraces (at least those that occur in the delivery room) or hypothermia on admission, and the ophthalmology team may have a separate system for tracking infants in need for a retinopathy exam, with variable input by frontline NICU staff. Therefore, when frontline staff responded to the survey, the processes associated with these outcomes may not have been at the forefront of their minds. Nevertheless, we wanted to test for correlations with these metrics, given that they had previously been identified as key metrics of NICU quality; knowing how these relate to safety and teamwork metrics is important information for leadership.

We did expect other subcomponents of the Baby-MONITOR to correlate with teamwork and safety climate. Specifically, based on the adult literature and clinical rationale, we expected NICU-level safety culture ratings to be associated with HAI, mortality, chronic lung disease, any human breast milk at discharge, and growth velocity. Each of these metrics requires multidisciplinary care teams to work together effectively, with flat hierarchies, good communication, and the freedom to speak up if an unsafe situation is identified.^24,25^ However, in this sample, the HAI rate was the only metric associated with safety and teamwork climate. It may be that the perceptions of unit teamwork and safety climate are associated with concrete behaviors that help prevent infections. For example, in one study, units with higher teamwork and safety climate ratings exhibited better hand hygiene.^8^

Potentially, the care processes linked with other outcomes are less concretely defined than with infection and not as readily recognizable to survey respondents. For example, growth velocity requires a more prolonged and complex set of interventions and has no sentinel outcome (e.g., infection). Although teamwork is critical in ensuring optimal nutritional support, providers may not as readily associate distinct behaviors with this outcome. We speculate that connecting actions and outcomes in the minds of providers may potentially be a critical intervention for improvement and one that could be monitored in its success through repeated assessments of safety culture and clinical outcomes. When these connections are not made, efforts to improve teamwork and safety behaviors may not directly result in better quality of care delivery.

The previous safety culture literature may also be subject to publication bias.^12,13^ Few randomized controlled studies exist to demonstrate causal relationships between safety culture and clinical outcomes. Thus, our findings are important and have practical implications. They add to a growing literature highlighting^11^ the fact that providers and managers need to be careful not to confuse efforts to improve safety culture with expectations for broad-based quality improvement. It is important to recognize that the evidence for links between safety culture and clinical outcomes is still being developed and that many things may influence clinical outcomes beyond what a safety culture survey can measure. Other studies have shown that interventions to improve teamwork (e.g., TeamSTEPPS) may improve teamwork behaviors without necessarily improving clinical outcomes.^25^ Although such activities may be necessary to create a favorable contextual environment for effective implementation of standardized evidence-based care delivery, they are not sufficient. Neonatal intensive care units still have to do the hard work of establishing care delivery mechanisms that optimize care outcomes. It they fail to do this, high safety culture ratings may merely reflect nice people providing suboptimal care on a range of outcomes.

Our results must be viewed within the context of the study design. Our cross-sectional study design is hypothesis generating. In addition, because all the analyses are conducted at the NICU level, our sample of 44 NICUs is relatively small to detect statistical significance, making the size and direction of the correlation coefficients more informative in this context. It is also important to understand that the strength of the correlations is not unusual with regard to institutional level variables. In a previous paper, we found just slightly higher correlations when we correlated clinical outcomes with one another.^26^ Here, we correlated dimensions (teamwork and safety climate), which are more distant in their relation to clinical outcomes. The correlations overall likely indicate that the Baby-MONITOR and dimensions of safety culture measure different aspects of quality of care delivery. By tracking both, institutions may gain insights about different components of service delivery that promote high-quality care and operational excellence.

Neonatal intensive care units participating in this study were not randomly chosen. Rather, they participated in the collaborative quality improvement effort for specific reasons. This may have introduced systematic bias into our analysis, the direction of which is not easily ascertained. Future studies will need to confirm our findings in larger samples and different healthcare settings. Without our knowledge, NICUs may have been engaged in a variety of quality and safety efforts that may have influenced respondent perceptions. In addition, the culture of safety survey information was gathered over the short timeframe of June 2011 through September 2011, which might not accurately reflect the safety culture scores throughout the three years used to evaluate the clinical outcomes (January 2010 to December 2012), potentially biasing our results toward the null. Any self-report survey may be subject to reporting bias, however, our relatively large sample size and response rate compare favorably with similar studies of safety culture assessments in the literature. In addition, we used some negatively valenced items, such as “In this NICU, it is difficult to speak up if I perceive a problem with patient care,” checked the psychometrics for this sample, and reported substantial variability between the NICUs.^19^ Finally, compared with other safety culture tools, the SAQ, and especially the safety and teamwork climate scales, perform favorably in terms of psychometrics, clinical applicability, and responsiveness to interventions.

## CONCLUSIONS

This study reveals significant correlation between HAI rates and NICU teamwork and safety climate. However, other metrics of quality predicted to correlate with teamwork and safety climate did not. Caution is needed in equating efforts to improve safety culture with expectations for broad-based quality improvement.
